# Development of a paper printed colorimetric sensor based on Cu-Curcumin nanoparticles for evolving point-of-care clinical diagnosis of sodium

**DOI:** 10.1038/s41598-022-09852-z

**Published:** 2022-04-15

**Authors:** Neeli Chandran, Prajit Janardhanan, Manikanta Bayal, Rajendra Pilankatta, Swapna S. Nair

**Affiliations:** 1 Department of Physics, Central University of Kerala, Periye, Kasaragod, Kerala 671316 India; 2 Department of Biochemistry and Molecular Biology, Central University of Kerala, Periye, Kasaragod, Kerala 671316 India

**Keywords:** Biochemistry, Biological techniques, Biotechnology, Materials science, Nanoscience and technology, Optics and photonics

## Abstract

The homeostatic control of Sodium (Na^+^) ion in the human body assumes paramount relevance owing to its physiological importance. Any deviation from the normal level causes serious health problems like hypernatremia, hyponatremia, stroke, kidney problems etc. Therefore, quantification of Na^+^ levels in body fluids has significant diagnostic and prognostic importance. However, interfering ions like Potassium ion (K^+^) is the major hurdle in sodium detection. In this work, we synthesized the clusters of 3–9 nm-sized highly stable and pure Copper nanoparticles surface functionalised with curcumin, through chemical reduction method. Each cluster of particles is encapsulated in a curcumin layer which is clearly visible in TEM images. The results show that these curcumin functionalized Cu NPs (CuC) are highly selective to the colorimetric detection of Na^+^. The ions like K^+^, Mg^2+^ and Zn^2+^ did not interfere with the Na^+^ in this sensing technique. Low-cost paper-based sensor strips are fabricated and calibrated for the sensing of sodium in the physiological range and shade cards were developed as a calorimetric guide for estimation of Na^+^ which makes them ideal point of care diagnostic platform. We demonstrate that the proposed CuC paper strip can be used for detecting Na^+^ concentration within the whole physiological range in both blood serum and urine.

## Introduction

Point of Care (POC) systems are the miniaturized platforms for portable, rapid, and cost-effective analysis and diagnosis in modern healthcare. It is a paradigm shift from the conventional clinical laboratory techniques to self or bedside diagnostic methods, which can be operated without experienced technicians. POC diagnostic devices offer qualitative and quantitative measurements on biomarkers and physiologically relevant components. Paper-based dipsticks, lateral flow assays, and printed electrodes have been widely used due to their easy manufacturing and convenient use^1,2^.

Sodium ion (Na^+^) is one of the physiologically relevant metal ions present inside the human body. The physiological range of sodium in blood level is 135–145 mM/l^3^. Deviation of the amount of Na^+^ ions in the human body can lead to severe health problems like hyponatremia, strokes, kidney problems etc. It can even increase the risk of cardiac issues, and hence the development of a sensor system for the detection of sodium has a high degree of clinical relevance^4^. Similarly, regular diagnosis of sodium concentration in excreted urine can help to detect the cardiovascular diseases and hypertension^5^.

For determining the sodium concentration in urine, different methods are used, including ion-selective electrodes^6^ and ion chromatography^7^, which are very precise and error-free methods. But the cost, total analysis time and sample required are relatively high. Alternatively, paper-based dipsticks are more convenient, cost-effective and give rapid results. In this context, different nanoparticle-based approaches are also in trend for the detection of biological sodium ion concentration. However, interfering ions are the main hurdle in the case of ion sensing. Ions like K^+^, Mg^2+^ and Zn^2+^ cause major interference in the case of sodium ion detection, which shows indistinguishable nature in colorimetric sensing.

The research on novel sensing technologies like lateral flow strips and microfluidic pads are making drastic changes in early diagnosis of diseases^8–10^. Several biomarkers specific to certain life-threatening diseases can be detected with the help of nano system based strip sensors or microfluidic devices^11^. Among the newly emerging sensing technologies, colorimetric sensing assumes much importance because it is an easy, cost-effective and reliable technique amenable to the visual detection. The presence of metal ions, proteins, amino acids and specific biomarkers can be detected using colorimetric sensing^12^. Also, paper-based strips are less expensive, and they are easy to use devices for patients as point of care diagnostic platforms^13,14^.

The instantaneous detection of analytes is possible by the change in color, which can be detected with the naked eye without any sophisticated instruments. Generally, the sensing mechanism is based on the molecular interaction between the specific analytes and the nanoparticle’s surface, which is functionalized with suitable surfactants. Nanoparticles, as label-free systems, exhibit efficient chemical or biological sensing properties. The availability of the finest colloidal metal nanostructures with precisely engineered surfaces makes the detection easier with high selectivity and sensitivity.

Metal nanostructures are excellent candidates in sensing, optical, and catalytic applications^15,16^. They have unique structural and optical properties, including quantum size effects, surface plasmon resonance (SPR), and large surface to volume ratio, which makes them ideal platforms in broad areas of material applications^17,18^. Recently, metal nanoparticles are being used for biomedical applications, including bioimaging^19,20^, biosensing^21^ and drug delivery^22^. Among metal nanoparticles, Copper nanoparticles (Cu NPs) are promising candidates for biomedical applications^23^, especially biosensing, owing to the SPR spectra in the visible range and fluorescence properties with favourable quantum yield^13,24,25^. Cu NPs are easily prone to surface oxidation, because copper oxides are more stable in the atmosphere. So, synthesis methods using efficient surface modification agents are more appealing to prevent the oxidation of Cu NPs^26,27^. The surfactants/ligands like citric acid^28^, glutathione^29^ and cysteine^30^, are generally used for protecting the surface of metal nanoparticles, especially Cu NPs. Therefore, surface modified Cu NPs hold great potential for the fabrication of colorimetric and fluorescence-based sensor strips^23,31,32^.

Here, we synthesized Curcumin functionalized Cu nanoparticles (CuC), and their application in metal ion detection is tested. Curcumin is the pigment extracted from turmeric. In this work, we used curcumin to protect the Cu NPs from oxidation and agglomeration. It is observed that the sensing system made up of CuC shows high levels of selectivity for sensing and quantifying Na^+^ ions. Paper-based sodium sensor strips have been fabricated, and visual detection of Na^+^ is possible in the physiological range using the same.

## Methods

Copper sulfate (CuSO_4_ 5H_2_O) with molecular weight 159.6 g/mol, and L-Ascorbic acid(C_6_H_8_O_6_) with molecular weight 176.1 g/mol were purchased from Sigma Aldrich. Sodium borohydride (NaBH_4_) and curcumin (Turmeric Yellow) (458-37-7) with molecular weight 368.39 g/mol were purchased from Sisco Research Laboratories Pvt. Ltd.

### Synthesis of curcumin assisted Cu NPs

Cu nanoparticles were synthesized by chemical reduction method using curcumin as the surface functionalizing agent. 0.5 g of curcumin was diluted in 5 ml of acetone. A solution of copper (II) sulfate pentahydrate (CuSO_4_·5H_2_O) (0.5 M) was made by dissolving the salt in 20 ml of de-ionized water. The metal salt solution was mixed with the curcumin solution and was kept under stirring for 10 min for homogenization. 20 ml aqueous solution of ascorbic acid (1 M) was added to the solution under constant stirring for 15 min, followed by dropwise addition of sodium borohydride (NaBH_4_) (5 M), which is the reducing agent. Ultrafine curcumin capped Cu nanoclusters were suspended in the solution. Aggregate clusters formed precipitate at the bottom of the beaker. The curcumin functionalized Cu NPs colloidal suspension (named as CuC) was decanted and used for further characterization and UV–Visible measurements.

### Procedures for colorimetric determination of Na^+^ ions

For the sensing and quantification of Na^+^, 50µL of CuC solution was added to 2 ml of varying concentration of Na^+^ ion solution (1 mM to 1 M) and the mixture was incubated at room temperature for 2 min. The absorbance of the sample was measured in the range 300–900 nm immediately. The selectivity towards Na^+^ ion was assessed by testing other cations (Ni^2+^, Mg^2+^, Cd^2+^, Co^2+^, K^+^, Sr^2+^, Zn^2+^, Ba^2+^and Ca^2+^).

### Preparation of test strips and colorimetric determination of Na^+^ ions

Cellulose based absorbent pad was used as test strips. The pad was cut into rectangular piece strips (0.5 × 3 cm) and each of the pieces were immersed into 1 ml of CuC solution. Finally, the strips (yellow in color) were dried at room temperature. 1 ml of the test solution was taken, and the test strip was immersed in it for 1 min. Subsequently, the color changes were visually observed to estimate the concentration of the Na^+^ in the sample. The selectivity of CuC paper test strip towards Na^+^ ion was assessed by testing other cations.

Images of the paper strips were captured using digital camera and analyzed in the RGB system using ImageJ software for the quantification of individual primary color contributions.

### Preparation of artificial urine sample

The artificial urine solution was prepared by following method described by Laube et al.^33^. This solution contained urea (25.00 g/L), NaCl (2.925 g/L), KCl (1.60 g/L), Na_2_SO_4_ (2.25 g/L), KH_2_PO_4_ (1.40 g/L), creatinine (1.10 g/L), NH_4_Cl (1.00 g/L), and CaCl_2_·H_2_O (1.103 g/L). All components were dissolved in deionized water. The pH of the solution was adjusted to 6. A set of artificial urine samples were prepared by changing the concentration of Na^+^.

### Biological sample analysis

10 mL of venous blood sample was drawn from each of the four healthy donors after 12 h overnight fasting. All blood samples were allowed to clot separately, at room temperature for 30 min. Following this, the blood serum was separated as a supernatant by centrifugation (2500 rpm for 15 min) at 4 °C, and the serum samples were stored at − 20 °C until further analysis. Further, 20 ml of urine samples were collected from healthy donors. The protocol was approved by the Institutional Human Ethics Committee of Central University of Kerala, Kasaragod (CUK/IHEC/2021/010). The blood sampling and processing was performed in accordance with their code of ethics and guidelines. The Institutional Human Ethics Committee waived the informed consent as the samples were taken from the investigators and is of minimal risk. All methods were performed in accordance with the relevant guidelines and regulations.

For the sensing and quantification of Na^+^, 50µL of CuC solution was added to 2 ml of blood serum (diluted) or urine sample, containing varying concentration of Na^+^ ion (10 mM to 250 mM) and the mixture was incubated at room temperature for 2 min. The absorbance of the biological sample (blood serum or urine) was measured in the range 300–800 nm after the addition. The test strip was immersed in the Na^+^ containing blood serum or urine for 1 min and the color changes of the strips were evaluated and the images were captured.

### Characterization

The optical properties like band gap, absorption maxima etc. of the Cu NPs were probed through UV-Vis spectroscopy (Model No. T60, Make-PG instruments Ltd.)^20^. The crystal structure of Cu NPs was studied using X-ray diffraction technique (Rigaku Miniflex) with Cu-K_α_ radiation (λ = 1.5406 Å)^34^. High Resolution Transmission Electron Microscope (Model JEM-2100, Make JOEL, accelerating voltage = 200 kV) was used to measure the particle/cluster size^34^. The elemental composition of the samples was determined using EDX (ZEISS GEMINI). Fourier transform infrared spectra (FT-IR) were recorded in the wavelength range of 4000 to 500 cm^−1^ to investigate the mechanism of conjugation (Model L160000U, Make- Perkin Elmer (Spectrum Two))^20^. The dynamic light scattering (DLS) measurements were performed (HORIBA, Nanoparticle analyzer SZ-100) to determine the hydrodynamic diameter and distribution of the CuC NPs in aqueous solution before and after the addition of sodium.

## Results and discussion

### Synthesis and mechanism of formation of CuC NPs

**Figure 1:**
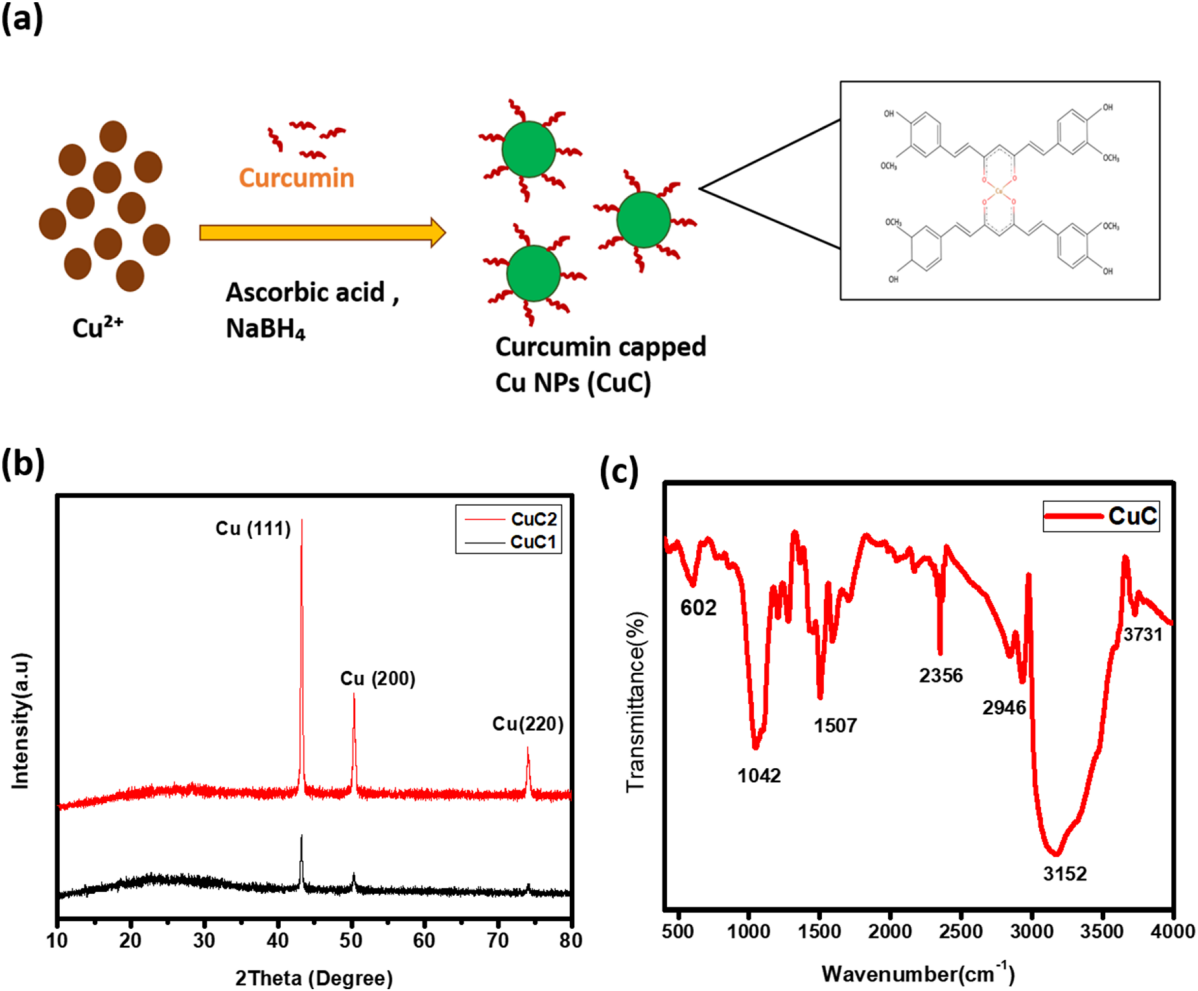
(**a**) The schematic illustration of formation of curcumin capped Cu NPs (CuC). (**b**) XRD pattern of curcumin assisted Cu NPs. (**c**) FTIR spectra of the sample.

Curcumin, the pigment derived from turmeric, is one of the most promising natural pigments. It has been reported that curcumin and related samples are cytotoxic to cancer cells and hence they are widely used in cancer drug delivery applications^35^. Curcumin can form strong complexes with metal ions. It contains α, β-unsaturated β-diketo moiety, which acts as an excellent chelating agent, that helps to form stable metal-curcumin complexes^35,36^. Here, we used 2:1(ligand: metal) stoichiometry which generally gives stable structures. The dark brown color precipitate was formed, which contains aggregates of curcumin capped Cu NPs. The colloidal suspension contains smaller CuC NPs of size less than 20 nm. Both are found to be highly stable for few months. This is because of the strong surface functionalization and chelating effect of curcumin. The proposed chemical structure of 2:1 curcumin-Cu is shown in Fig. 1a. Attachment of curcumin onto the surface of Cu is through the two enolic groups, and the metal ion is replaced by enolic proton, and the *o*-methoxy phenolic moiety in the curcumin remains flawless in the complexes.

### Characterization of structural and optical properties of CuC

#### Structural analysis

The typical XRD pattern of curcumin assisted Cu NPs (CuC) are shown in the Fig. 1b, which shows the formation of pure metallic Cu with crystallinity, with an intense peak at 2Ө = 43° oriented along the (111) plane. The other peaks indexed at (200) and (220) planes, correspond to 2Ө = 50.32° and 74.10°, respectively. There are no peaks of unreacted sulfides and copper oxides. The result is consistent with the cubic Cu standard spectrum with space group Fm-3 m (225) (ICDS No. 01-070-3038). The samples CuC1and CuC2 were synthesized with different concentrations of CuSO_4_.

FTIR analysis was performed to detect the functional groups and characteristic bond formation between Cu and curcumin. Ascorbic acid and curcumin are the organic reagents which are involved in the covalent bond formation. Figure 1c shows the FTIR spectra of CuC. The specific attachment of curcumin on the surface of Cu NPs, was assessed by comparing the FTIR spectra of curcumin (Suppl. Fig. S1) and that of the curcumin stabilized Cu NPs. Curcumin exhibited its signature peak at 3508 cm^−1^ which is attributed to the –OH stretching vibration. The peak of benzene stretching vibrations are observed at 1589 cm^−1^. C=O and C=C stretching vibration peaks are observed at 1506 cm^−1^. The sharp peaks are observed at 1428 cm^−1^ and 1279 cm^−1^ which is due to the olefinic C–H bending vibrations and the aromatic C–O stretching vibrations, respectively.

The IR absorption peaks observed at 607 and 633 cm^−1^ for plain curcumin is shifted to 602, and 676 cm^−1^ for the curcumin capped Cu NPs. These peaks correspond to the CH vibration of the aromatic ring in the curcumin molecule. A peak present in the IR spectrum of pure curcumin at 804 cm^−1^, that disappears for Cu NPs, hints towards the interaction between curcumin on the surface of Cu NPs. The peak observed at 1151 cm^−1^, which is attributed to the in-plane vibration of CCH, is shifted to 1203 cm^−1^. A new peak is observed at 1704 cm^−1^ which can be due to the binding of Cu with the C=O group of curcumin. After the binding of curcumin with Cu, the metal–oxygen bond was characterized by IR spectroscopy and a characteristic peak is observed at 1629 cm^−1^ corresponding to the carbonyl peaks in the complexes, which showed a slight shift in wave number with lower intensity because of the coordination to metals^37^. A sharp elongated peak that is not observed for curcumin is found at 2350 cm^−1^, indicating the presence of curcumin Cu complex. The strong IR absorption peak observed at 2946 cm^−1^ for Cu NPs can be attributed to the CH stretching of –OCH_3_ or CH_3_ is indicative of the presence of curcumin on Cu NPs. A broad peak observed at 3150 cm^−1^ can be ascribed to O–H stretching vibrations due to the OH functional groups present in ascorbic acid and curcumin. Therefore, the FTIR spectra analysis confirmed that the curcumin is conjugated to the Cu NPs.

The elements present in the synthesized Cu NPs were assessed by Energy Dispersive X-ray (EDX) analysis as depicted in Suppl Fig. S2. The spectrum consists of the peaks corresponding to the elements C, O and Cu. The carbon content estimated can be due to the carbon tape used in the SEM analysis. The presence of Oxygen can be due to the adsorbed moisture. The strong intense peaks observed in EDX correspond to copper, which proves the formation of copper nanoparticles.

**Figure 2:**
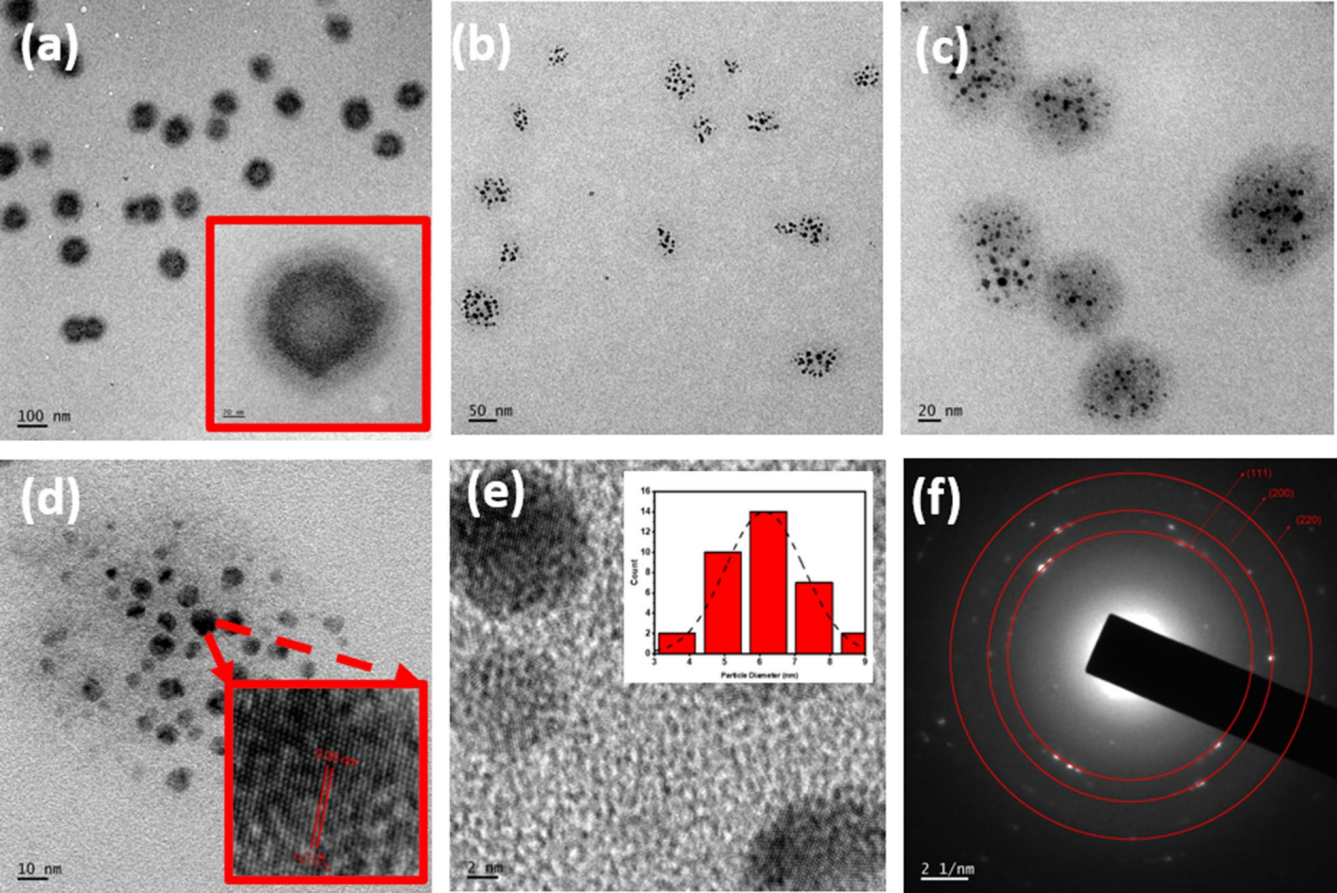
(**a**) TEM image of curcumin capped Cu NPs (CuC). Inset figure shows the curcumin cage and inner core. (**b**) and (**c**) Clusters of Cu NPs embedded in the curcumin with different magnifications. (**d**) A cluster of Cu NPs. Inset figure shows HRTEM image (**e**) A single particle with approximately 8 nm size. Inset figure shows particle size distribution in a cluster of CuC. (**f**) SAED pattern of the sample.

TEM images of colloidal CuC samples are shown in Fig. 2. Curcumin acts as a cage, and a cluster of Cu NPs are embedded inside this cage. Figure 2a shows that each cluster have ~ 100 nm size, and smaller Cu NPs are placed in it. Curcumin caged Cu NPs with different magnifications are shown in Fig. 2a–c. The Cu NPs are crystalline, and they show clear lattice fringes. The sizes of the Cu NPs are approximately 6 nm. The particle size distribution inside the cage is shown in the Fig. 2e. The particles size varies from 3 to 9 nm.

In the HRTEM images, an interplanar spacing of 0.18 nm is observed as shown in Fig. 2d, which correspond to the (111) plane of Cu. Further, the HRTEM images also provide the proof for the formation of single crystalline curcumin modified Cu NPs. Curcumin cage protects Cu NPs from oxidation. It can be concluded that the novel fabrication process developed here is quite successful in the formation of pure crystalline curcumin caged Cu NPs. Selected Area Diffraction pattern of the sample is shown in Fig. 2f. Crystalline dots corresponding to the planes (111), (200) and (220), are clearly observed in the diffraction pattern.

#### Optical analysis

**Figure 3:**
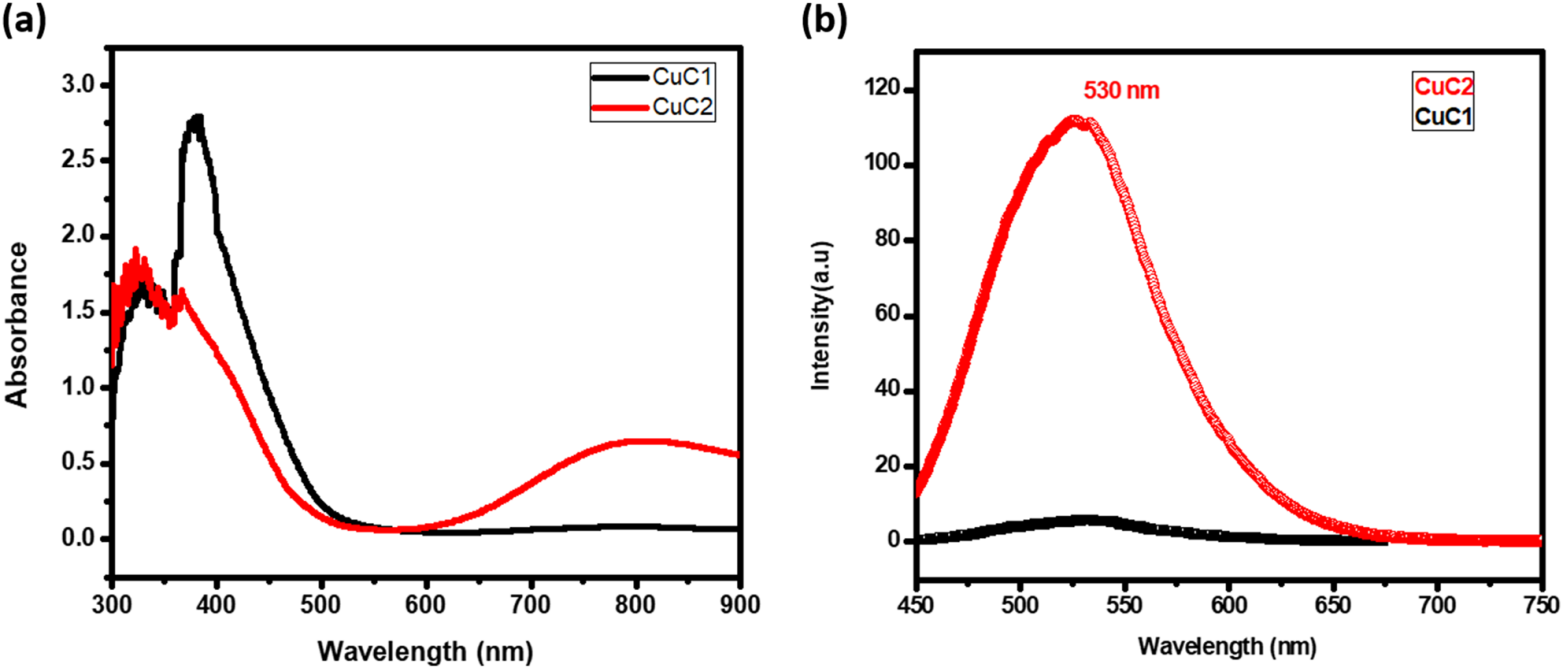
(**a**) UV–Visible spectra and (**b**) Photoluminescent emission spectra of CuC.

The UV–Visible spectra of CuC are provided in Fig. 3. The broad peak noted at 400 nm is due to the characteristic band edge transition peak of CuC and the value of absorbance reached a minimum at 490 nm. A broad and less intense peak centred at 803 nm, which is the characteristic SPR peak of Cu, is found to be shifted to higher wavelength region^18^. This is due to the bulk effect of Cu NPs cluster embedded on curcumin. The concentration of Cu precursors was varied by fixing the other parameters, including the concentration of curcumin which is shown in Fig. 3a. It is observed that the SPR peak intensity was enhanced by increasing the concentration of Cu. In the case of larger particles, SPR peaks shift towards longer wavelengths. Here, each big cluster of Cu NPs might have induced bulk SPR effect.

Fluorescence spectra of CuC showed an emission peak centred at 530 nm under the excitation of 400 nm (Fig. 3b). Cu NPs exhibits the band-to-band emission in the blue wavelength region. It is shifted towards 530 nm due to the influence of curcumin which has small-scale fluorescence around 500 nm. In the sample CuC2, a high intense broad emission peak is observed at 530 nm, which contains a higher concentration of Cu. At the same time, the intensity of emission spectra of CuC is increased by increasing the excitation wavelengths. The emission spectra of CuC with varying excitation wavelengths are shown in Suppl Fig. S3.

The intensity obtained is the maximum at 420 nm excitation wavelength. No significant shift is observed in the emission spectra with respect to changing excitation from 350 to 420 nm.

### Detection of Na^+^ ions using CuC

#### Design principles and mechanism of colorimetric assay

**Figure 4:**
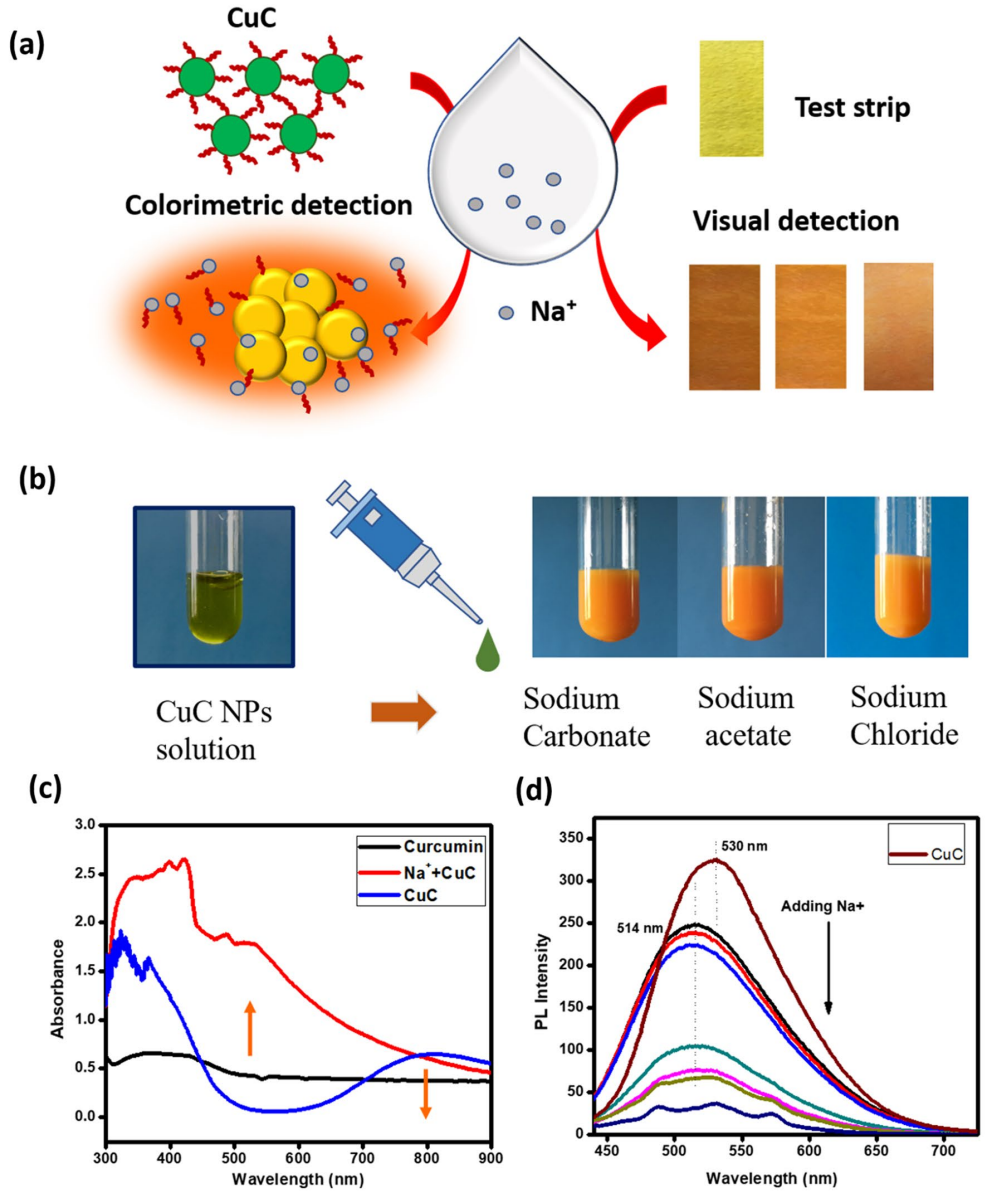
(**a**) Schematic representation showing the mechanism of the selective determination of Na^+^ using CuC based colorimetric probe (Image concept courtesy^38^). (**b**) Colorimetric detection of Na^+^ from different sources. (**c**) Absorbance spectra of CuC NPs solution before and after the addition of Na^+^. (**d**) Fluorescent emission spectra of CuC by the addition of different concentration of aqueous solution containing Na^+^.

The design principle of the colorimetric assay is shown in Fig. 4a. The sensing mechanism can be explained based on the chemical reaction between the curcumin molecules and Na^+^, which leads to the formation of aggregates of Cu with Na-curcumin complexes. The brick orange color is due to the formation of Cu–Na-curcumin complexes which is shown in the Fig. 4b. Here, CuC was added to the solutions containing varying concentrations of Na^+^. Figure 4c shows the changes in the absorbance spectra after the addition of Na^+^.

The complex formation suppresses the SPR and the SPR absorption peak at 803 nm is completely vanished and a new broad absorption peak is appeared at around 557 nm. Bare curcumin did not exhibit any prominent absorbance peak in the visible range, and hence the new broad peak is originated due to the formation of stable Na–Cu-Curcumin complexes. The surface protecting ligand (curcumin) is released from the surface of Cu (0) upon the addition of Na^+^, leading to the aggregation of cores and forms larger particles. As a result, the SPR peak is disappeared, and the absorption band edge is red-shifted towards 500 nm.

The fluorescent spectra of CuC under the excitation of 420 nm exhibited an emission peak at 530 nm, which is due to the presence of curcumin on the surface of Cu NPs. However, after the addition of Na^+^, the fluorescent spectrum became broader, intensity is decreased and the peak is slightly shifted to 514 nm. The fluorescent intensity also showed a significant decrease after the addition of Na^+^, as shown in Fig. 4d. By adding Na^+^ solution, the basic characteristics of Cu NPs are diminished.

Due to the significant color change, qualitative visual detection is easily possible. But the quantification of Na^+^ in the test samples is more relevant. Paper test strips produced by drop-casting of the CuC NP suspension, was used for the sensitive determination of Na^+^ through the shade cards showing calibrated color change (from yellow to orange) in the presence of Na^+^.

#### Mechanism

##### FTIR analysis

Pure curcumin has IR bands in the fingerprint region which is clearly shown in Suppl Fig. S1. The FTIR spectra of CuC before and after the addition of Na^+^ (in different concentration) are shown in Suppl Fig. S4(a). The peaks of curcumin at 602 cm^−1^ remains the same with increased intensity in Na–CuC complex. It is observed that, a new peak is emerged at ~ 870 cm^−1^ with the increase in concentration of Na^+^, showing the signatures of the complexation of Na^+^ with CuC. This is due to the CuC–Na complex. The peaks correspond to the C–O–C stretching vibrations and benzene stretching vibrations observed at 1042 and 1589 cm^−1^ are shifted to 1089 and 1608 cm^−1^, respectively in Na–CuC complex. The intensity of the peak corresponds to the C–O–C stretching vibrations at 1089 cm^−1^ observed in Na–CuC complex, showed a gradual decrease in intensity with the increase in concentrations of Na^+^. A peak at 1360 cm^−1^ became broader and more elongated upon the addition of Na^+^. A new broad peak is clearly seen at 1645 cm^−1^ in CuC–Na complex, which can be assigned to the stretching vibration of the aromatic ring in the curcumin molecule. This is the evidence of the interaction between Na^+^ and CuC (Suppl Fig. S4(b)). It is also observed that an increase in the molecular interactions of Na^+^, resulted in an increase in the intensity of the particular peak. A sharp peak of CuC at 2350 cm^−1^ which indicates the presence of curcumin on Cu NP surface, is disappeared after the addition of Na^+^ which provides the signatures of complexation with Na^+^.

##### DLS analysis

The increase in the hydrodynamic diameter of CuC upon getting complexed with Na^+^, is confirmed through DLS. The study supports the theory of formation of aggregates with the addition of Na^+^, which prompted for a red shift in the absorption edge. Uniformly distributed ultra-small sized Cu particles are observed in the CuC solution, while an increase in the hydrodynamic diameter is observed for CuC + Na^+^ complex. From the figure (Suppl Fig. S5(a)), it is evident that the CuC particles possess a hydrodynamic diameter of 4–5 nm. However, upon introduction of Na^+^ ions in to CuC, the hydrodynamic diameter has been increased to ~ 100 s of nms, The observations obtained through the DLS measurements are in good agreement with the previous reports on colorimetric sensing^39,40^. The frequency of smaller sized Cu NPs is reduced with increase in the concentration of Na^+^ due to the formation of aggregated compounds. When intensity of Na^+^ increases, more Cu NPs are made complexes with Na^+^ (Suppl Fig. S5).

##### Mechanism of Na^+^ detection

By the addition of CuC into Na^+^ ion solution, first, it forms a complex with Na^+^, and from the DLS studies, it is evident that Na^+^ induces cluster growth in proportion to the Na^+^ concentration which resulted in a linearly increasing absorption coefficient at 550 nm. The charge barrier around the CuC complex also influences the absorption. At very high concentration of Na^+^, due to the positive charge cloud around the CuC complex (Suppl Fig. S4(c)), further cluster growth is inhibited. As evidenced from the FTIR analysis, the peak that appeared at 2350 cm^−1^ is found disappeared, and the complex with Na^+^ produced a deep-brown color, after which the color is getting saturated which is indicative of the highest detection limit of the sensor (which is much above the clinically relevant range).

#### Evaluation of the feasibility of CuC based colorimetric assay

**Figure 5:**
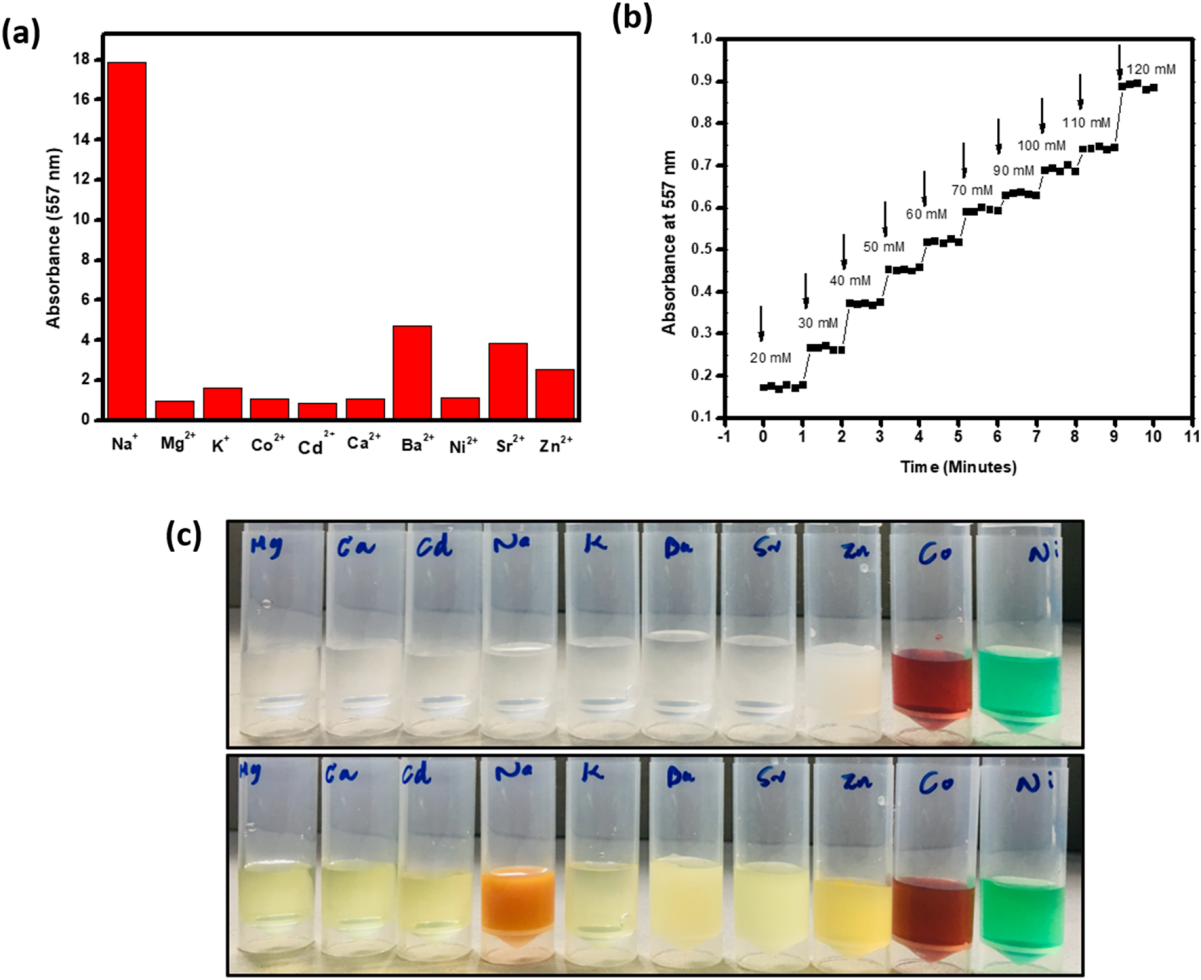
(**a**) Absorbance response of CuC solution in the presence of different metal ions in aqueous solution. (**b**) The absorbance vs time plot of Na^+^ in different concentrations (20–120 mM). (**c**) The photograph of different metal ions before and after adding CuC colorimetric sensing probe solution.

To study the selectivity of the colorimetric assay for Na^+^, absorbance spectra were recorded by adding sensing solution (CuC) into various metal ions independently in the same condition. As shown in Fig. 5a, we investigated the response of Na^+^ and other metal ions including Ni^2+^, Mg^2+^, Cd^2+^, Co^2+^, K^+^, Sr^2+^, Zn^2+^, Ba^2+^and Ca^2+^ ions. The Fig. 5a indicates that there is no significant response observed for other metal ions, suggesting high degree of selectivity for Na^+^ detection. K^+^ is the most possible interfering ion, which is almost similar to Na^+^ in size as well as properties. However, sensor system under the current investigation showed preferential selectivity to Na^+^.

The stability of CuC is shown in Suppl Fig. S6(a). where the absorbance spectra were recorded after two months of its synthesis. Slight changes in the intensity of the SPR peaks are observed after two months. The absorbance peaks do not show any significant shift in wavelength. This ensures the stability and long shelf life of CuC sensing solution.

This shows the strong affinity of Na^+^ ions to the ligand, curcumin. Stability as well as selectivity of the sensor system is essential in the case of analytical sensors. The absorbance of CuC at 557 nm, after the addition of Na^+^, was recorded up to 16 min which shows the stability of the signal detection. After 2 min, the signal remains stable, which is shown in Suppl Fig. S6(b). So, after adding each set of samples into the CuC solution, it was incubated for 2 min at room temperature. The absorbance was recorded in every 20 s up to 1 min. This kinetics measurement provided stable values in each concentration. Absorbance vs time plot gives the stability of the CuC colorimetric sensing probe system which is shown in the Fig. 5b. The photograph of color changes, before and after the addition of CuC colorimetric probe solution, is shown in Fig. 5c.

#### Analytical performance of the colorimetric Na^+^ assay

**Figure 6:**
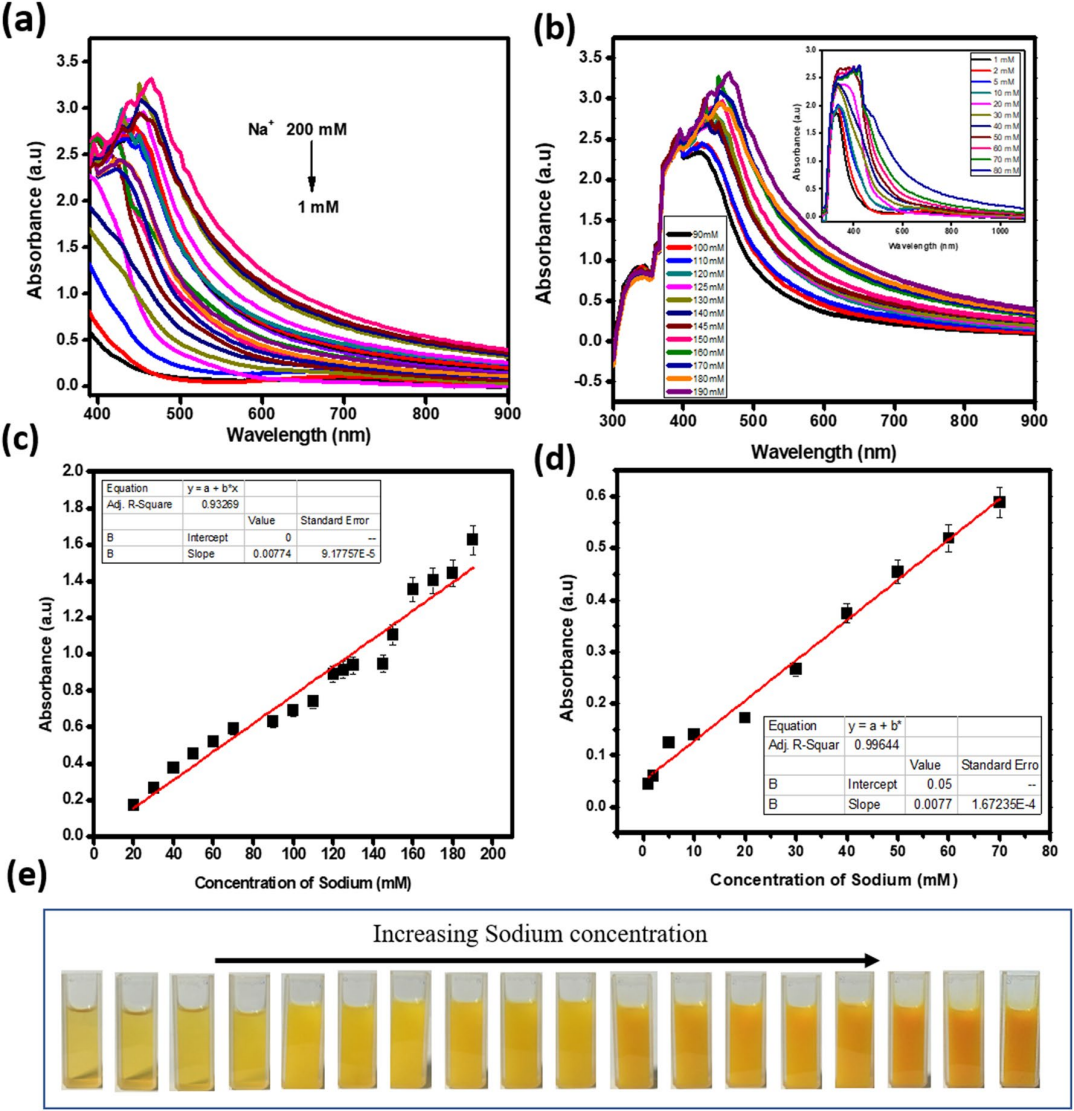
(**a**) Absorbance profile of CuC upon the addition of Na^+^. (**b**) Concentration of Na^+^ from 90 to 190 mM. Inset figure- concentration of Na^+^ from 1 to 80 mM. (**c**) and (**d**) Linear responses of absorbance changes at 557 nm with respect to the concentration of Na^+^. (**e**) Photograph of CuC with different concentrations of Na^+^. Error bars represent standard error of the mean (n = 5).

High sensitivity is another important criterion of the development of an efficient sensor. The titration experiment is performed to evaluate the sensitivity of the proposed colorimetric assay. Figure 6a gives the gradual enhancement of absorbance spectra by the addition of Na^+^ in aqueous medium. In the absorbance spectra, a peak near 500 nm is enhanced upon the addition of Na^+^. The absorption band shifted to longer wavelength region with the decreasing intensity of the peak at 803 nm. Figure 6b also depicts the absorbance spectra with concentrations ranging from 90 to 190 mM. The inset figure shows the concentration of Na^+^ from 1 to 80 mM. The spectra exhibit a new peak at 460 nm, and the whole spectra has an enhancement in intensity by the stepwise addition of Na^+^. In lower concentrations (1–10 mM), a small SPR peak of CuC is observed, which is shifted to 720 nm and the same is disappeared at higher concentrations of Na^+^. Figure 6c shows linear response of absorbance (at λ = 557 nm) towards increasing concentration of Na^+^ (from 20 to 200 mM; slope = 0.0077, R^2^ = 0.932). Figure 6d depicts the absorbance (at λ = 557 nm) at lower concentration range (1–80 mM; slope = 0.0077, R^2^ = 0.996). The sodium concentration in human samples, like urine and blood serum, are in the range of 20–250 mM. For a Na^+^ ion sensor to perform properly in environmental or biological samples, it should be able to work in these biologically relevant concentrations. So, we selected this range of concentration for the detection of Na^+^. Figure 6e shows the color change observed by the addition of Na^+^ ions into the CuC colorimetric probe solution. The lowest detection limit calculated from the Fig. 6c, based on 3σ/slope, is 65 × 10^–6^ M. For the detection of Na^+^, it is established from the current investigation that CuC solution is an ideal colorimetric sensor with high selectivity and sensitivity, low LOD and naked eye recognition potential.

#### Fabrication of paper test strips for visual determination of Na^+^ ions

**Figure 7:**
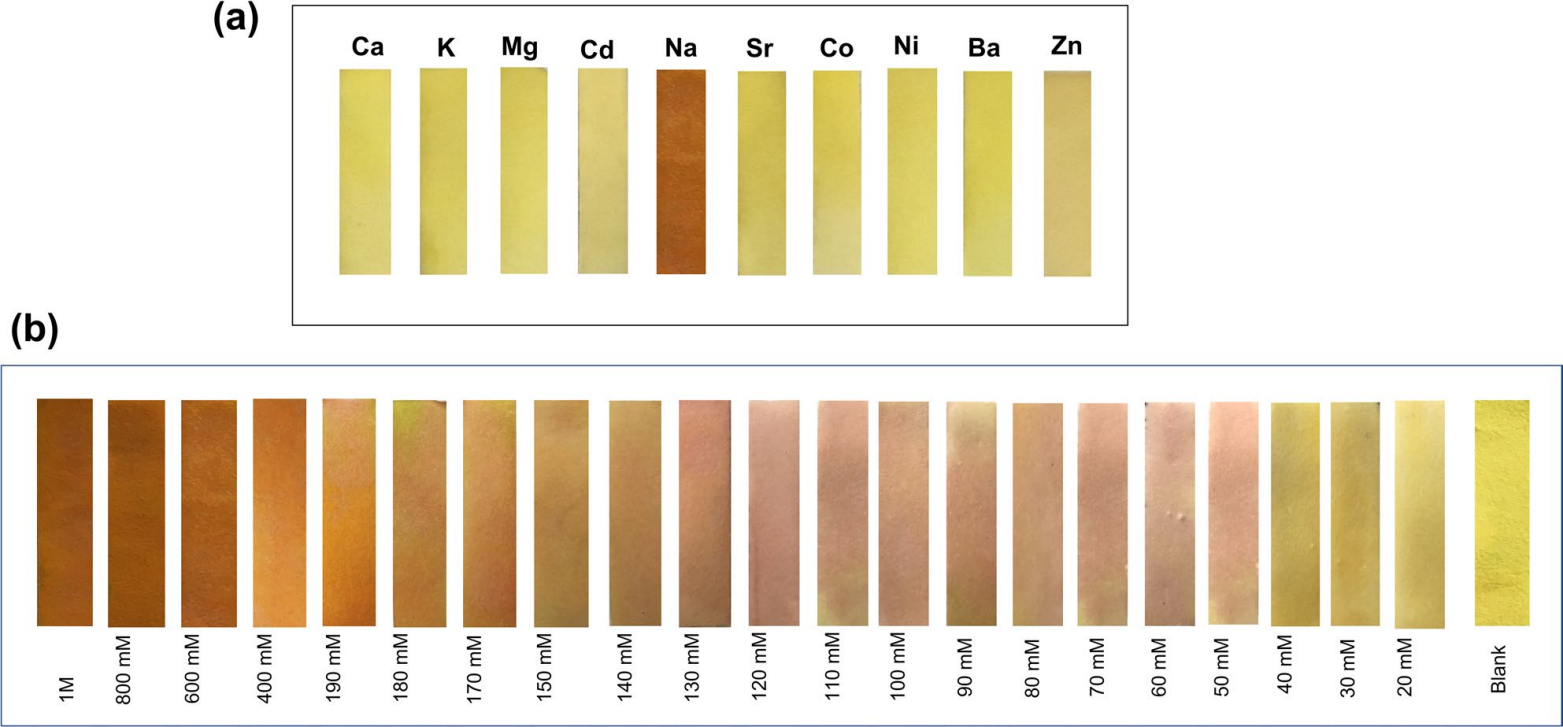
(**a**) Selective visual colorimetric response of Na^+^. Photograph of CuC paper test strips exposed to different metal ions. (**b**) Photographs of CuC based test strip with increasing concentration of Na^+^ from 20 mM to 1 M exhibiting color changes.

Test strips were fabricated by soaking the cellulose-based absorbent pad into CuC colorimetric probe solution for 30 s followed by drying at room temperature. Compared with the blank pad, it is light yellow in color, as shown in the Fig. 7. Test strips based visual detection methods are eco-friendly, low cost and are easier to use. The strips were stored at 4 °C for three months to see their shelf life, and it is observed that the sensor system is very stable with high shelf life and hence can be employed to detect the sodium concentrations in human samples. The test strips are highly selective against Na^+^ which is clearly shown in the Fig. 7a. It is observed that the color of the test strips changed from yellow to pale pink and then to orange with the increasing concentration of Na^+^. Figure 7b shows the photograph of test strips with increasing concentration of Na^+^ from 20 mM to 1 M. So, this proves the excellent selectivity of Na^+^, as compared to the other metal ions, especially K^+^ which is the most interfering ion for Na^+^. Therefore, CuC-based colorimetric test strips can offer a convenient and low-cost method to the sensitive detection Na^+^ in human samples with high degree of selectivity even in the presence of K^+^.

In order to probe into the mechanism of Na^+^ sensing, Cu NPs are synthesized without curcumin. Copper nanoparticles (Cu NPs) alone (without curcumin) also showed sensing potential. After the addition of Cu NPs, color of Na^+^ solution is changed from clear to brick orange color. However, bare Cu NPs are found to be less stable against surface oxidation. Hence, it can be concluded that the Cu NPs contribute to the sensing part, and the role of curcumin is to stabilize the Cu NPs so that sustained signal read out is possible in the CuC complex system with a higher shelf life for the sensor. Curcumin also takes part in the complexation with Na^+^ inducing cluster growth which in turn resulted in a linear variation in the absorption intensity with respect to the increase in Na^+^ concentration. (Na^+^ promotes cluster growth in bare Cu NPs too.) However, Cu-Curcumin-Na^+^ complexation helps in obtaining a stabilized signal with linearity in absorption due to the surface protection of Cu NPs. Sensing potential of plane curcumin also is tested. There is no linearity with respect to the increasing concentration of Na^+^ in the visible range. With increase in the concentration of Na^+^, a systematic color variation could not be observed in the visible range. The absorbance spectra of samples (Na^+^ solutions) with the addition of Cu NPs and curcumin solution separately are shown below (Suppl Fig. S7).

For comparison, color changes of paper strips using Cu NPs, curcumin, and CuC after the addition different concentration of Na^+^ also shown in Suppl Fig. S8(a). Cu NPs solution (without curcumin) is light green in color, and after dropping and drying the Cu NP solution, paper strips found to be colorless. After the addition of Na^+^ solution into these strips, the paper strip exhibited an orange color. But the paper strips of curcumin didn’t show any significant color change (although a mild color change is observed, it is constant in the detection range, and no linearity is observed in the presence of varying concentration of Na^+^). But CuC shows gradual color change in the presence of sodium. Color intensity of the digital images of test strips are quantified using RGB (Red, Green, Blue) analysis, which is the most common color analysis method for colorimetric quantification. The plot of RGB analysis of digital images of the paper test strips of Cu NPs, Curcumin, and CuC in the presence of different concentration of Na^+^ are shown in Suppl Fig. S8(b). It confirms the colorimetric linearity in CuC with increasing concentration of sodium.

#### Determination of Na^+^ ion in simulated urine samples

Sodium acts as both an electrolyte and a mineral that keeps the fluid balance and electrolyte balance in the human body. The intracellular and extracellular fluid levels are very important for the human body, which helps in proper functioning of muscles and nerves. This also helps to maintain stable blood pressure levels. In the case of athletes, mental and physical fitness is related to the electrolyte balance in the body, which can be determined by the sodium levels in sweat.

Insufficient sodium level inside the body is known as hyponatremia, which occurs when the water and sodium levels are out of balance. The normal sodium level in blood should be between 135 and 145 mEq/L. Sodium levels below 135 mEq/L is known as hyponatremia. The sodium excreted through urine gives an indication of kidney diseases, hyponatremia, the function of adrenal glands etc. Furthermore, hypernatremia is another common problem because of higher levels of sodium in the blood. Kidney diseases, uncontrolled diabetes, dehydration etc. are co-factors of hypernatremia. To identify the imbalances of sodium, urine tests are the easiest way. The paper strip based colorimetric method is the most efficient and easiest method to diagnose routine sodium concentration in urine and sweat.

**Figure 8:**
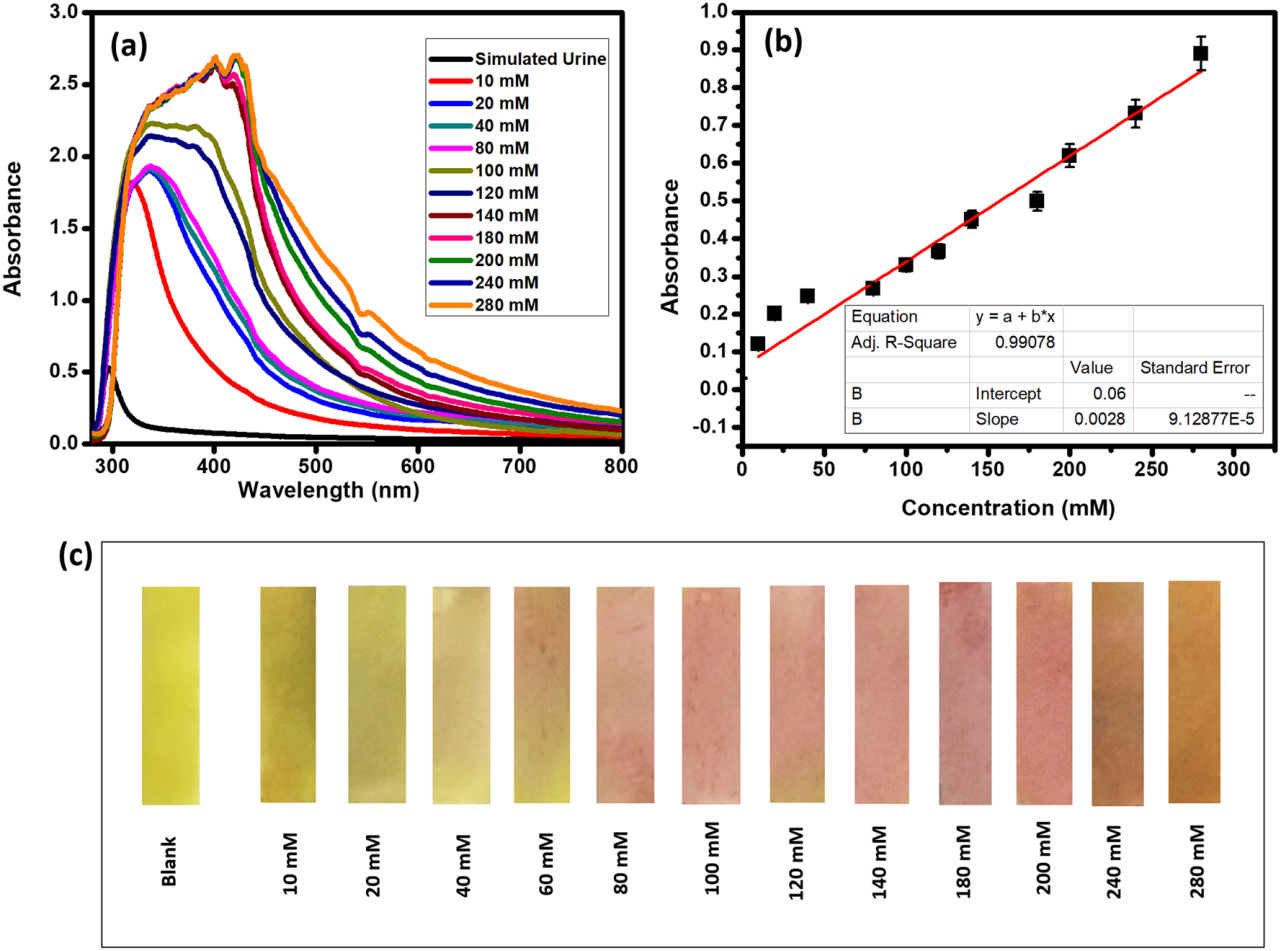
(**a**) Absorbance profile of simulated urine samples upon the addition of CuC. (**b**) Linear responses of absorbance changes with respect to the concentration of Na^+^ in the simulated urine samples. (**c**) Photograph of CuC-test strips with different concentrations of Na^+^ containing urine samples. Error bars represent standard error of the mean (n = 5).

Here, we prepared the simulated urine with urea, sodium, potassium, and creatine in reported physiological range. Different concentrations of Na^+^ are added into the samples so as to simulate both the hyponatraemic and hypernatraemic conditions. Interestingly, CuC colorimetric probe solution was found to detect the presence of Na^+^ in simulated urine samples. Figure 8a gives the absorbance spectra of simulated urine with different concentrations of Na^+^ ions from 5 to 260 mM after the addition of CuC sensing solution. The curve fitting (Fig. 8b) shows a linear response towards the increasing concentration of Na^+^ from 10 to 280 mM with slope = 0.0028 and the regression coefficient, R^2^ = 0.990. Lower value of slope is a signature of slower response. This is due to the availability of other large molecules like urea, creatine etc. in the sample, which affects the speed of migration of cations and hence the response will be slower as compared to the plane aqueous solution.

The linear absorption response with respect to the Na^+^ content in the urine sample is observed, and the same is depicted in Fig. 8b. This reveals that the color changes and absorbance spectra induced by CuC and Na^+^ are totally independent with respect to other contents in urine like urea, chlorides, and potassium. The lowest detection limit was calculated from Fig. 8b, based on 3σ/slope, which is equal to 97 × 10^–6^ M. The photographs of CuC test strips with different concentrations of sodium-containing urine samples are shown in the Fig. 8c.

The urine and blood serum contain sodium levels in the range varying from 20 to 200 mM. CuC colorimetric probe exhibits a high degree of selectivity and sensitivity in this range. So, this is an efficient diagnostic platform for sensing Na^+^ levels in human samples.

#### Determination of Na^+^ ion in biological samples

**Figure 9:**
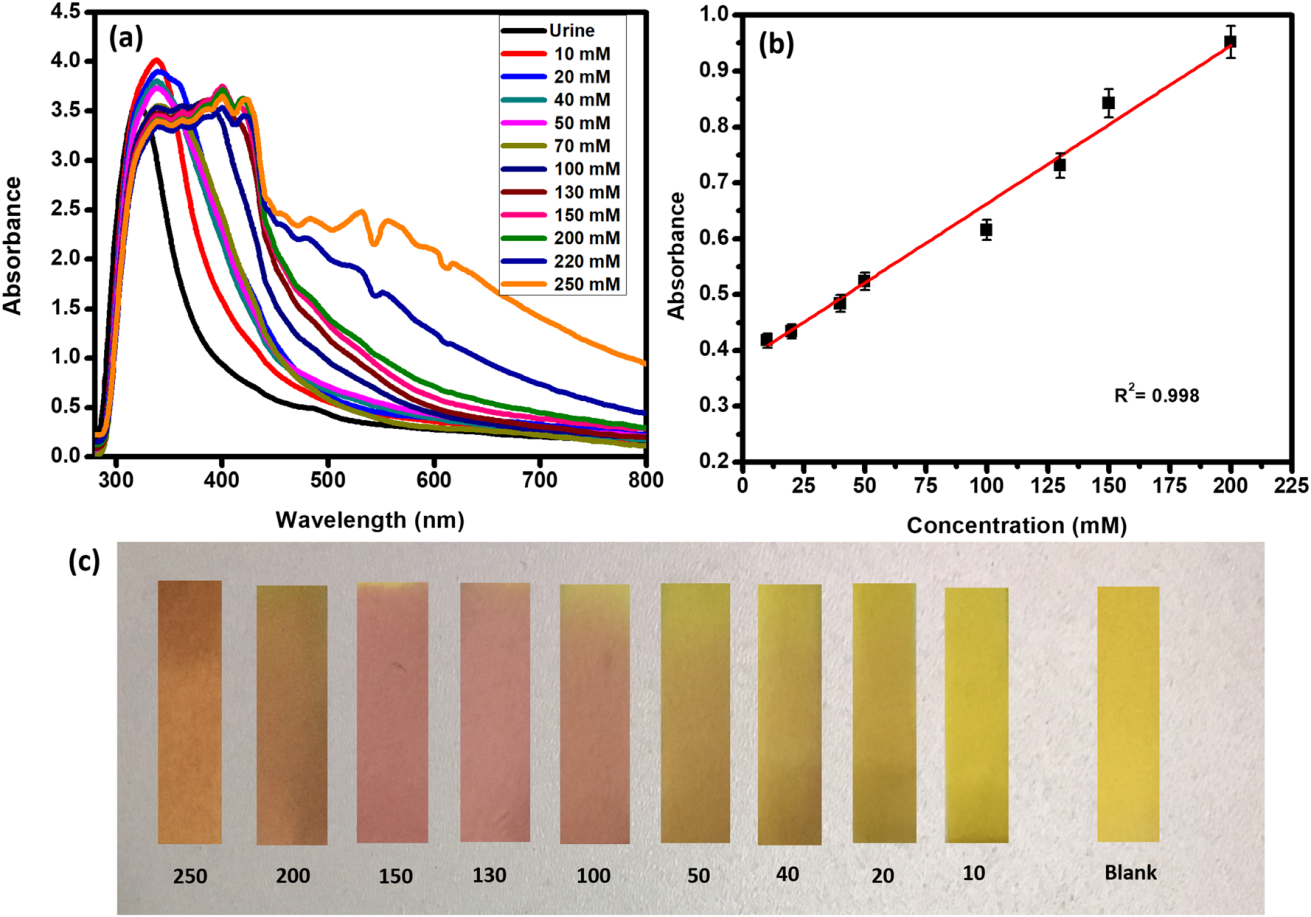
(**a**) Absorbance profile of biological urine samples upon the addition of CuC. (**b**) Linear responses of absorbance changes with respect to the concentration of Na^+^ in the simulated urine samples. (**c**) Photograph of CuC-paper test strips with different concentrations of Na^+^ containing urine samples. Error bars represent standard error of the mean (n = 5).

Figure 9 depicts the results of Na^+^ detection in biological urine. The absorbance of the samples was measured using UV–Visible spectra. It can be seen that by increasing the concentration of Na^+^, the corresponding absorbance increases proportionally. A linear response was obtained at a fixed wavelength, 557 nm. The R^2^ value of the regression line was obtained as 0.998. Increasing the concentration of Na^+^ from 10 to 250 mM within the physiological range led to the increase in the absorbance. The CuC coated paper strips shows color changes from yellow to pink and then to orange by increasing the concentration of Na^+^.

**Figure 10:**
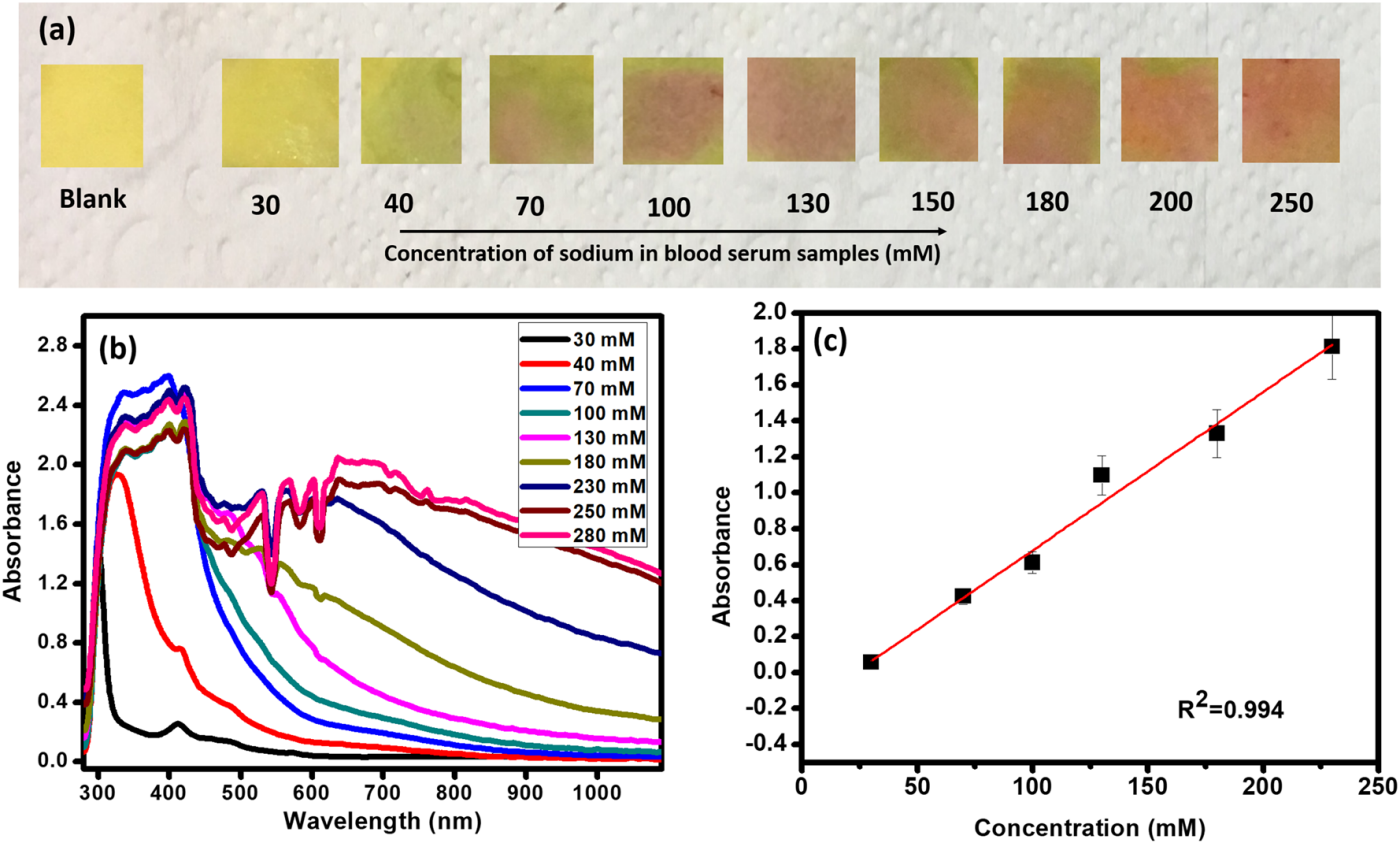
(**a**) Photograph of CuC-paper test strips exposed to blood serum samples with varying concentrations of Na^+^. (**b**) Absorbance spectra of blood serum samples upon the addition of CuC sensing solution. (**c**) Curve fitting showing the linear responses of absorbance Vs Concentration at 557 nm. Error bars represent standard error of the mean (n = 5).

The fabricated CuC paper strips are used for the detection of Na^+^ in the human blood serum. Figure 10a shows a specific linear color change in the CuC paper strips with respect to the increasing Na^+^ concentration similar to the test tube-based Na^+^ sensing results. Absorbance spectra shows linear response with the increasing concentration of Na^+^ in blood serum samples at fixed measurement conditions (Fig. 10b,c). Other biological components and ions present in the blood serum did not show interference with the sensing of Na^+^.

**Figure 11:**
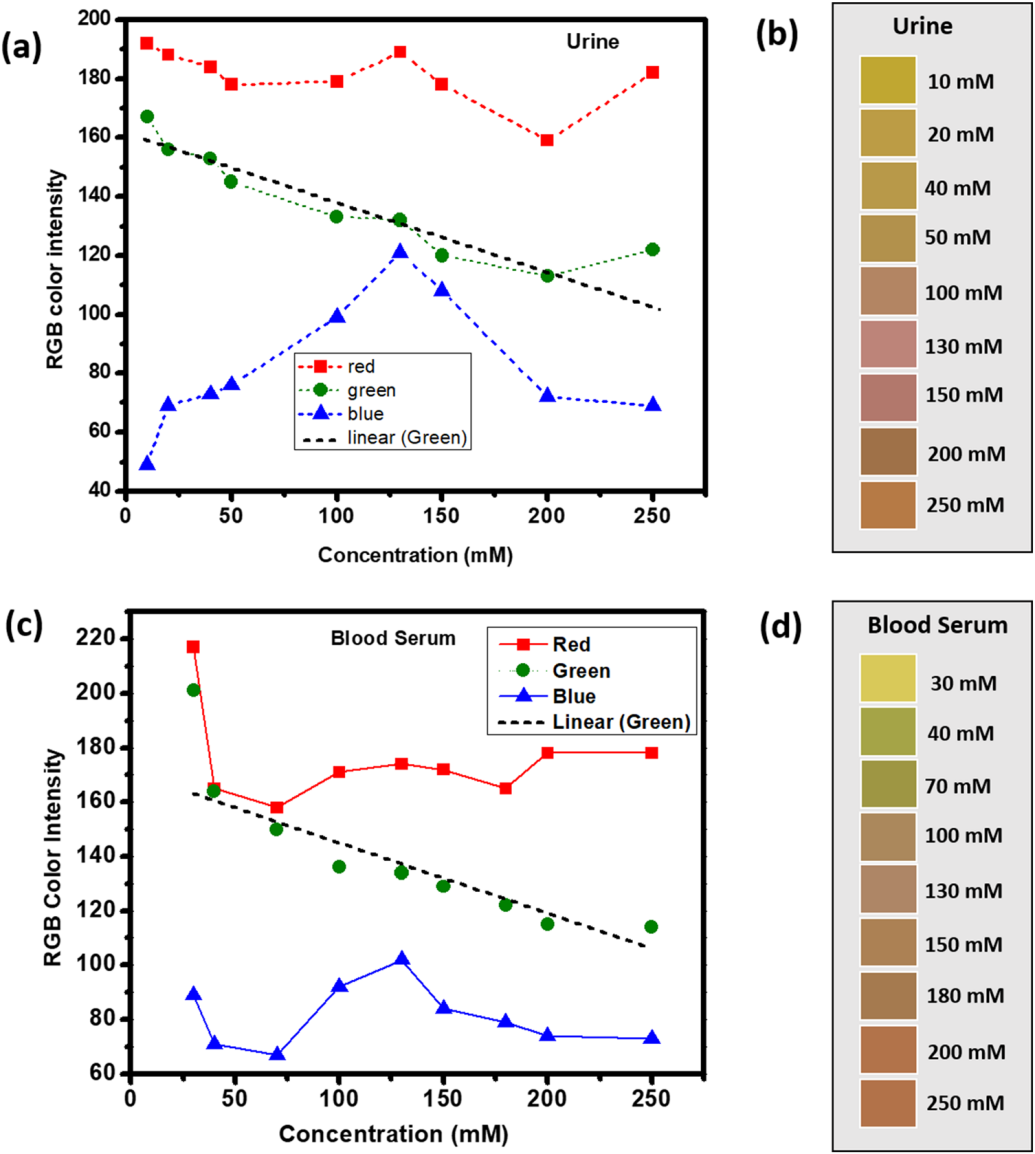
The plot of RGB analysis of digital images of the paper test strips (**a**) urine and (**c**) blood serum. Corresponding shade cards representing Na^+^ concentration (**b**) urine and (**d**) blood serum.

The individual analysis of the plot of R, G, and B color channel intensities in both serum and urine (Fig. 11a,c), showed a linear correlation of green channel intensity with respect to the concentration of Na^+^. Corresponding color shade cards (Fig. 11b,d) are developed which can be used as a direct sensing guide for the patients and thus serves as an efficient point of care diagnostic tool for the estimation of sodium in both blood serum and urine.

## Conclusion

Variation of sodium concentration in blood and urine are the diagnostic indicators of diseases resulting from the imbalance in sodium homeostasis of the body. Therefore, continuous monitoring of Na^+^ concentration is helpful in these contexts. Here, we fabricated a strip based low-cost colorimetric sensing method using curcumin capped Cu NPs. The sensor gives a linear absorption response towards the sodium concentration in the physiological range. The sensor is calibrated for the known Na^+^ concentrations as shade cards which can be used for the estimation of Na^+^ concentration in test samples. So, the physiological fluctuation in sodium concentration can be effectively monitored using the sensor test strips developed with high degree of cation selectivity and sensitivity. The platform can be used as a low-cost point of care diagnostic tool.

## Supplementary Information

Supplementary Information.
