# Supplementary material for: Development of a paper printed colorimetric sensor based on Cu-Curcumin nanoparticles for evolving point-of-care clinical diagnosis of sodium
Source: Sci Rep. 2022 Apr 15;12:6247. doi: 10.1038/s41598-022-09852-z (PMC9012761; doi:10.1038/s41598-022-09852-z)
Supplement: Supplementary file 1 — Supplementary Information. [file 41598_2022_9852_MOESM1_ESM.docx]

**Electronic Supplementary material.**

**Development of a paper printed colorimetric sensor based on Cu-Curcumin nanoparticles for evolving point-of-care clinical diagnosis of Sodium**

**Neeli Chandran^a^; Prajit Janardhanan^b^; Manikanta Bayal^a^ ; Rajendra Pilankatta^b^ ; Swapna S Nair^a^***

*^a^ Department of Physics, Central University of Kerala, Periye, Kasaragod, Kerala, India, 671316.*

*^b^ Department of Biochemistry and Molecular Biology, Central University of Kerala, Periye, Kasaragod, Kerala, India, 671316*.

* Corresponding author

E mail address:[swapna.s.nair@gmail.com](mailto:swapna.s.nair@gmail.com)





Fig.S1. FTIR spectrum of pure curcumin powder


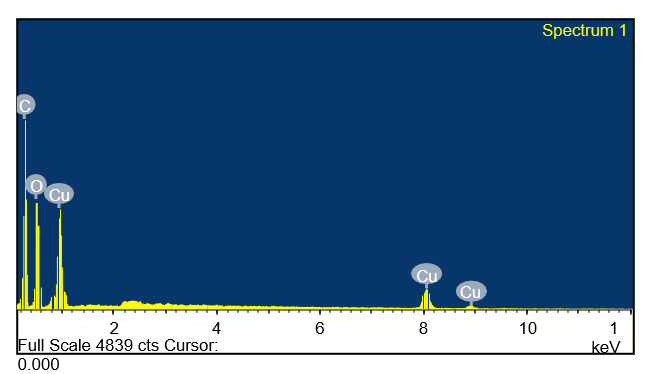


Fig.S2. EDX spectrum of the sample.


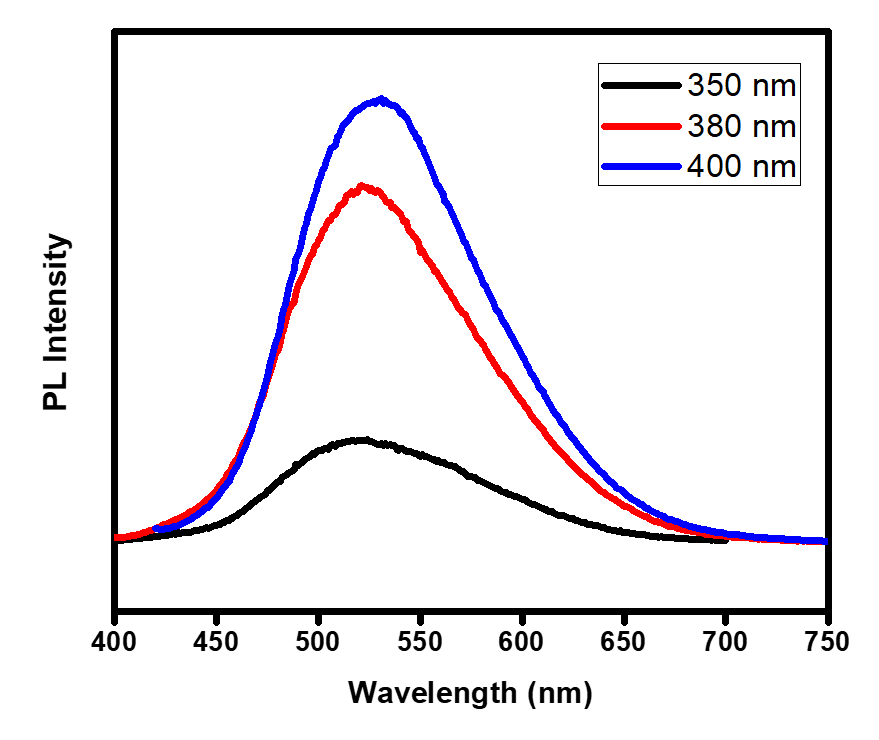


Fig. S3. Photoluminescent emission spectra of CuC with different excitation wavelengths.


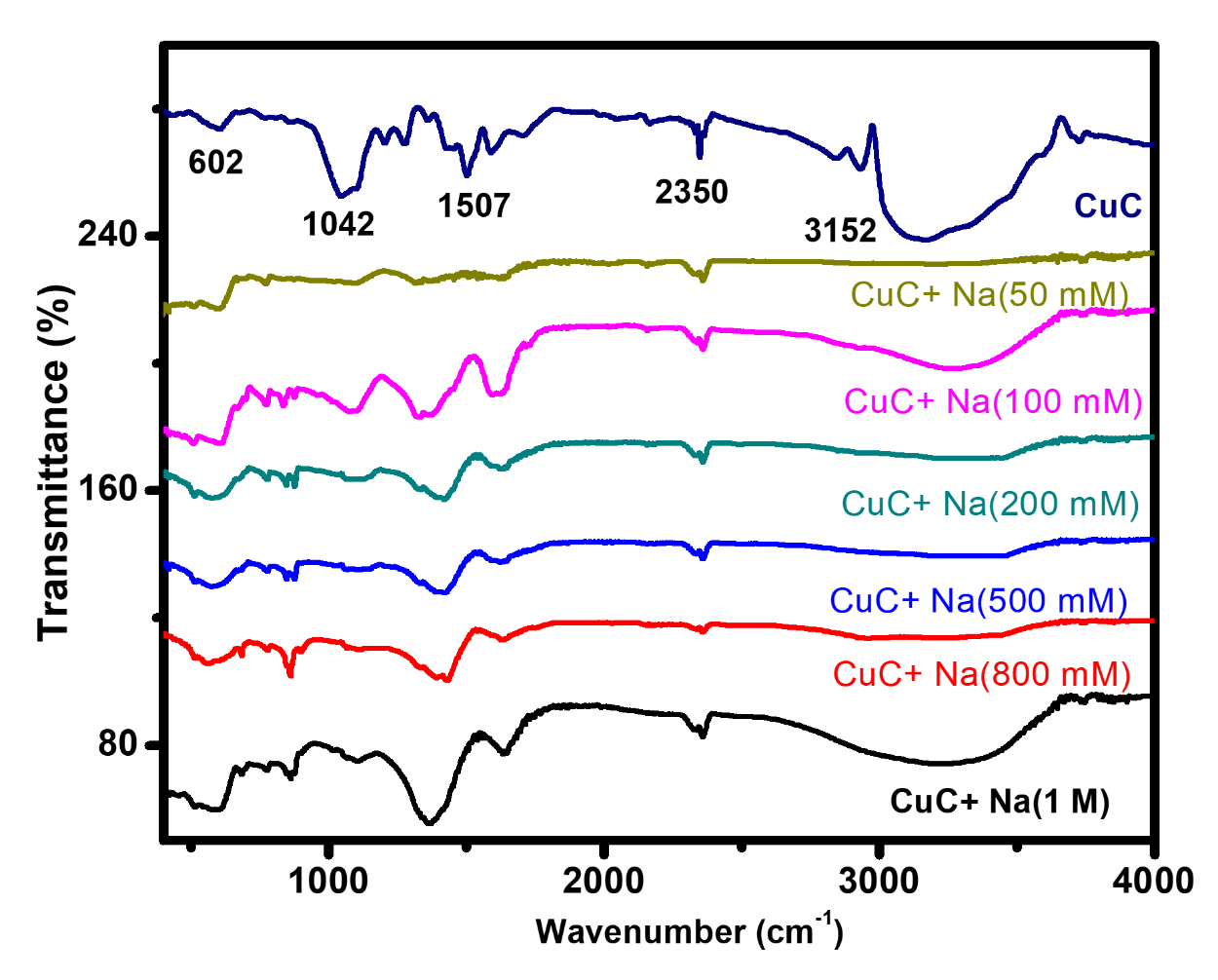


**(a)**

**(b)**


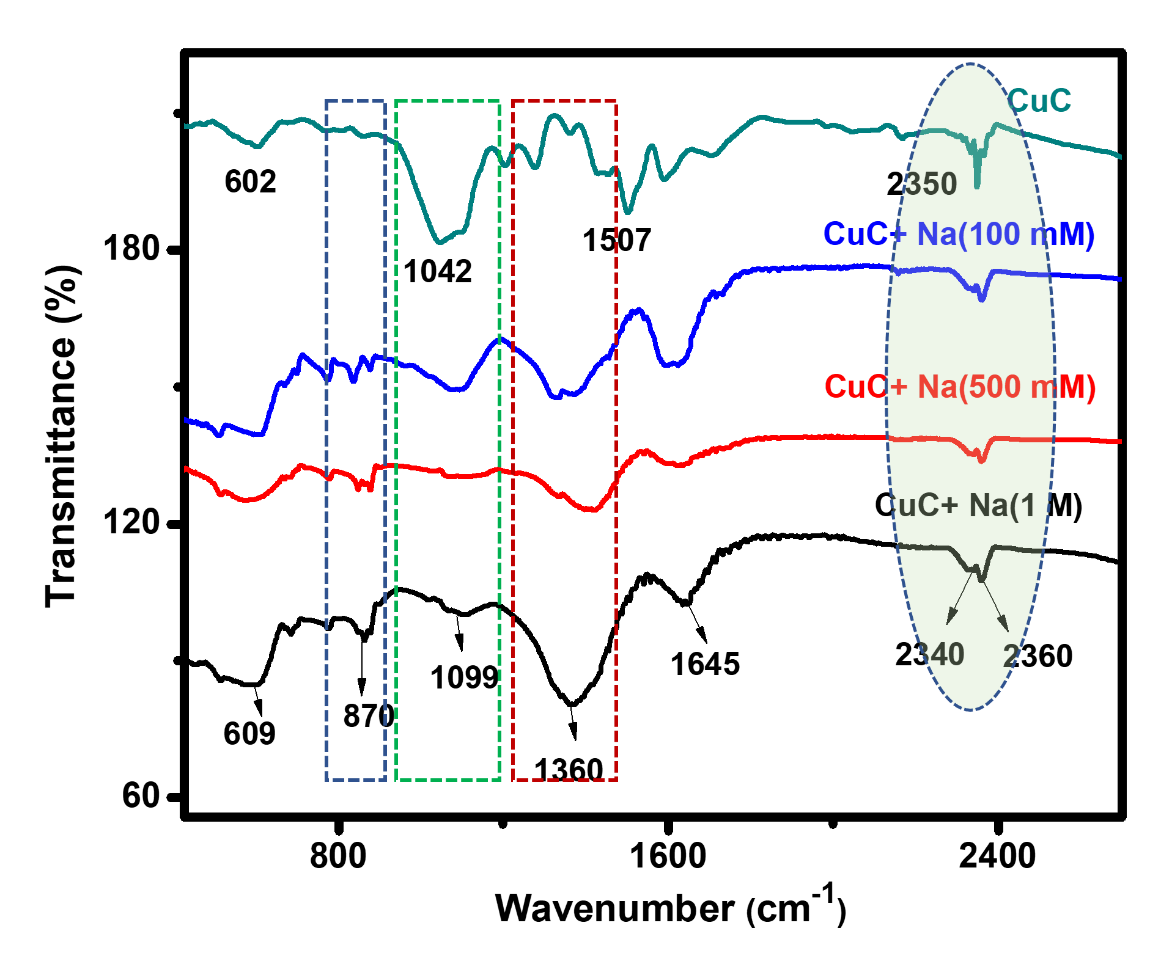


**(c)**


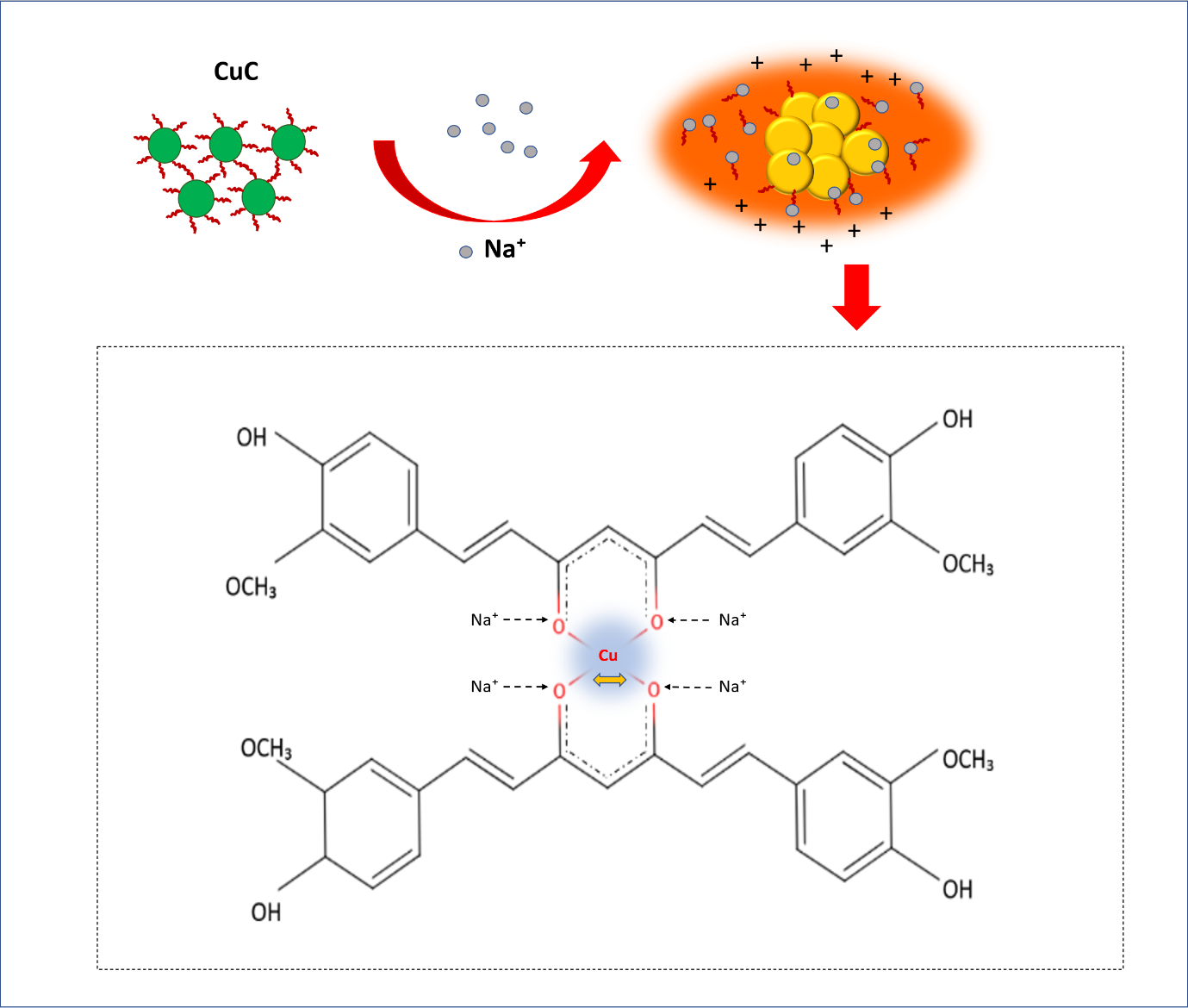


Fig.S4. (a) FTIR spectra of CuC+Na^+^ complexes in different concentrations of Na^+^. (b) Zoom of the spectra for better visualization of the changes in the peaks between 400 and 2700 cm^−1^. (c) Proposed structure of CuC complex after the addition of Na^+^


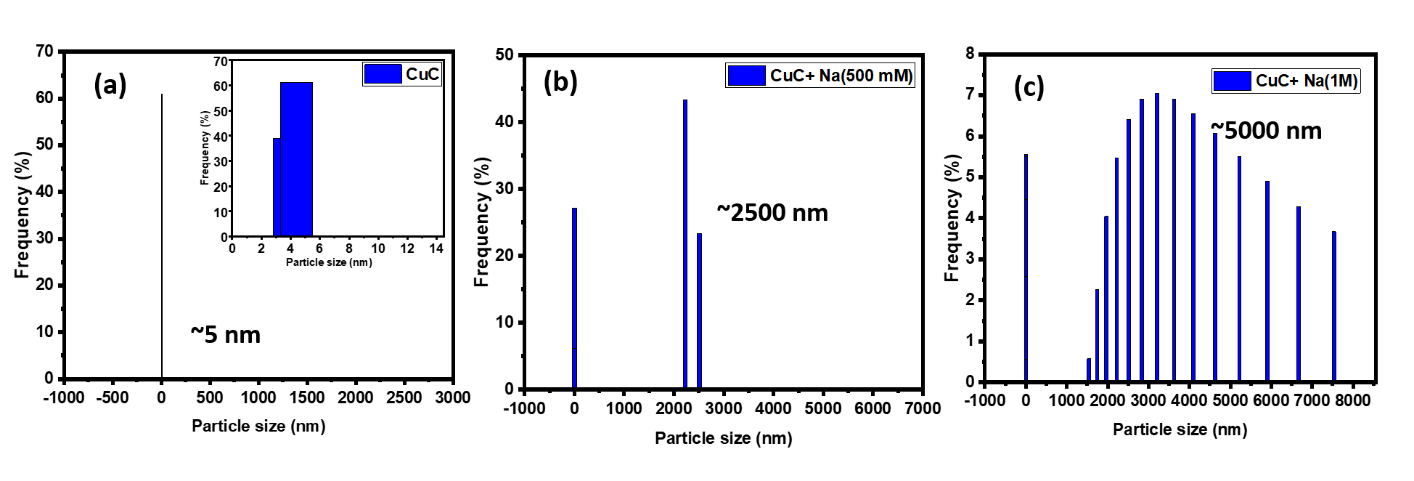


Fig.S5. DLS measurements of (a) CuC, (b) the CuC with Sodium (500 mM) and (c) CuC with sodium (1M) showing the hydrodynamic diameter before and after the addition of sodium.


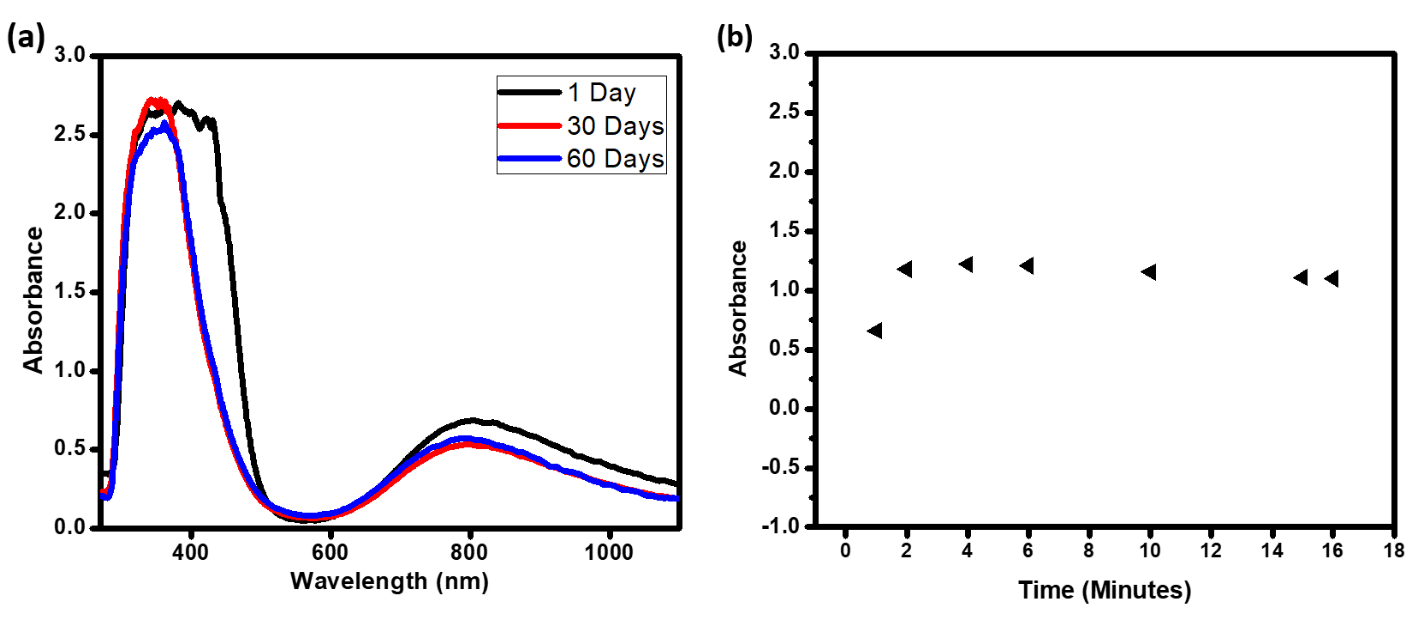


Fig.S6(a) The stability of CuC (b). The absorbance of CuC after adding Na^+^ up to 16 minutes.


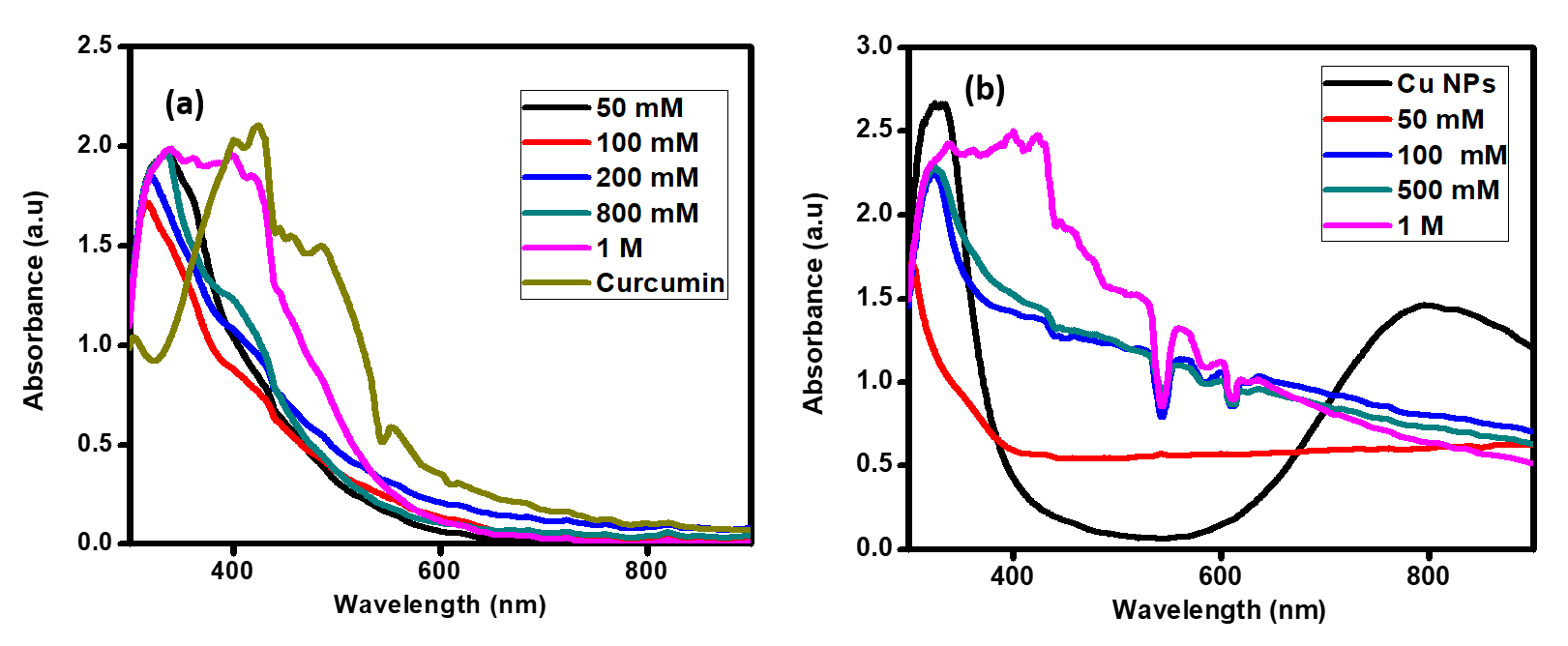


Fig.S7: Absorbance spectra of (a) curcumin and (b) Cu NPs in the presence of different concentrations of Na^+^.


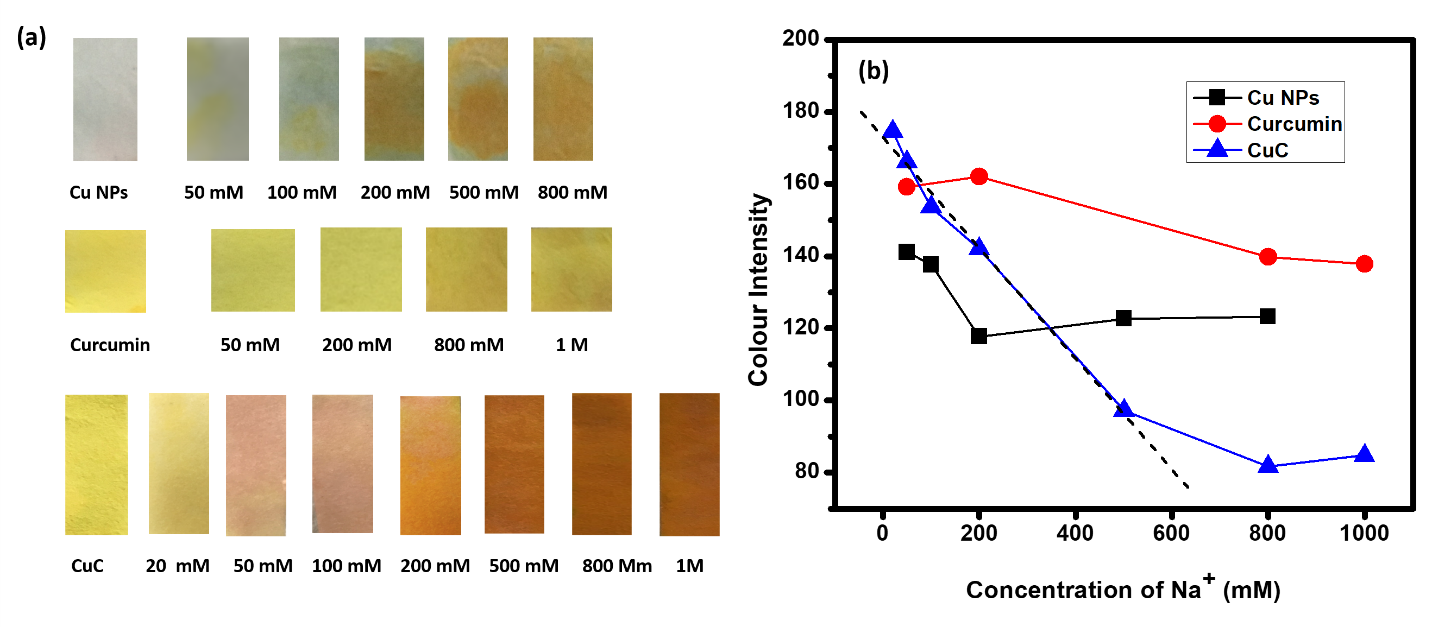


Fig.S8: (a)Color changes of paper test strips of Cu NPs, curcumin, and CuC in the presence of different concentrations of Na^+^. (b) RGB analysis of the paper strips with respect to the concentration of Na^+^.
